# Association between the BDNF Val66Met polymorphism and major depressive disorder: a systematic review and meta-analysis

**DOI:** 10.3389/fpsyt.2023.1143833

**Published:** 2023-06-21

**Authors:** Yuxia Wang, Ou Li, Nana Li, Zhongwei Sha, Zhenghao Zhao, Jian Xu

**Affiliations:** Department of Mental Diseases, Shanghai Municipal Hospital of Traditional Chinese Medicine, Shanghai University of Traditional Chinese Medicine, Shanghai, China

**Keywords:** BDNF Val66Met, polymorphism, major depressive disorder, systematic review, meta-analysis

## Abstract

**Study objectives:**

This meta-analysis analytically reviewed recent studies concerning the potential associations between the brain-derived neurotrophic factor (BDNF) Val66Met polymorphism and susceptibility to major depressive disorder (MDD), with subgroup analyses for race and age.

**Methods:**

Relevant case-control studies were systematically searched for in PubMed, Embase, the Web of Science, China National Knowledge Infrastructure (CNKI), Wanfang, and Sinomed databases. A total of 24 studies were finally identified to have reported outcomes including alleles, dominant genes, recessive genes, homozygosity, and heterozygosity. Subgroup meta-analyses were performed based on participant age and ethnicity. Publication bias was represented by funnel plots. All meta-analyses of the randomized controlled trials included for evaluation were performed using RevMan5.3 software.

**Results:**

The findings revealed no significant association between BDNF Val66Met polymorphism and MDD. However, the Met allele was found to be associated with genetic susceptibility to MDD among white populations on subgroup analysis (OR = 1.25, 95% CI: 1.05–1.48, *P* = 0.01). In the genetic model, dominant (OR = 1.40, 95% CI: 1.18–1.66, *P* = 0.0001), recessive (OR = 1.70, 95% CI: 1.05–2.78, *P* = 0.03), and homozygous (OR = 1.77, 95% CI: 1.08–2.88, *P* = 0.02) genes were all associated with MDD.

**Conclusions:**

Despite the outcome limitations, this meta-analysis confirmed that the BDNF Val66Met polymorphism is a susceptibility factor for MDD in white populations.

## 1. Introduction

Major depressive disorder (MDD), also termed clinical depression, manifests with low mood, anhedonia, and fatigue; cognitive decline, autonomic nervous function changes, sleep disturbances, and appetite changes are also frequently reported (1, 2). MDD is an affective regulatory psychiatric disorder characterized by significant and persistent feelings of depression. It is a common and frequently encountered disease (3). It is a prevalent and disabling mood disorder that poses a significant burden to society. The etiology of MDD is complex and multifactorial, involving genetic, neuroendocrine, and sociopsychological factors. It is widely accepted that MDD arises from a complex interplay between these factors, which ultimately contribute to alterations in brain structure and function, leading to the development of depressive symptoms (4, 5).

Recently, brain-derived neurotrophic factor (BDNF) gene polymorphisms have been reported to play important roles in depression and cognitive impairment (6, 7). As the most abundant neurotrophic factor in the central nervous system, BDNF is involved in both developmental and regulatory functions such as plasticity. Abundant in brain areas key to emotional regulation such as the hippocampus, hypothalamus, amygdala, and neocortex, BDNF functions to support cholinergic, dopaminergic, and serotonergic signaling (8, 9). The BDNF genotype was reported to be closely associated with the development of MDD (10). Furthermore, the Val66Met polymorphism was found to be associated with genetic predispositions to anxiety and depression in animals (11).

The BDNF gene is located on human chromosome 11p13. Of several known relevant gene polymorphisms, Val66Met has attracted the greatest interest. This non-synonymous G to A single-nucleotide polymorphism is found at position 196 of exon 2 and results in the substitution of methionine for valine (Val → Met) at codon 66 (12, 13). Although patients suffering depression are considerably more likely to be Met allele carriers, no definitive association has been proven to date (14, 15). To what extent Val66Met associates with susceptibility to MDD thus warrants investigation.

## 2. Materials and methods

### 2.1. Database search

On 10 September 2022, the Embase, PubMed, Web of Science, China National Knowledge Internet (CNKI), Wanfang, and Sinomed databases were searched for relevant literature using keywords as follows: (major depressive disorder OR depression disorder OR depression disease) and (brain-derived neurotrophic factor) and (Val66Met OR RS6265) and (polymorphism OR mutation OR variation OR genotype OR gene). Literature in both English and Chinese was searched.

### 2.2. Selection criteria

All studies included in our meta-analysis conformed to the following inclusion criteria: (a) the study focused on the relationship between MDD and Val66Met; (b) the study was a case-control study published in the form of a treatise; (c) the study included both MDD patients and normal controls; (d) study controls were healthy persons; (e) study sample sizes were adequate and allele and genotype frequency data were available; (f) the study applied the diagnostic criteria of DSM-IV or DSM-V or CCMD-3 or HDRS≥18 or ICD-10 MDD; and (g) the study employed rigorous experimental methods for detecting genetic polymorphisms. For duplicate publications, the study with the greater sample size was included in our analysis.

### 2.3. Data extraction

All studies selected for analysis were sequentially screened on the basis of title and abstract content; studies which did not meet our selection criteria were excluded while duplicates were removed. The following data were extracted using pre-defined data extraction tables: first author's name; year of publication; country of study; study subject ethnicity; sources of both case and control groups; sample sizes; case and control group genotypes; allele frequencies; allele and genotype identification methods; whether Hardy-Weinberg equilibrium (HWE) conditions were met; and diagnostic criteria. Data evaluation was performed independently by Ou Li and Yuxia Wang; inconsistencies were addressed via research team discussions.

### 2.4. Statistical analysis

Data were analyzed using Revman 5.3 software. The odds ratio (OR) was used as a measure of BDNF gene polymorphism in MDD and a 95% confidence interval (CI) was considered. The χ^2^ test was used to evaluate heterogeneity among results of each study. When I^2^ ≤ 50%, studies were considered to have been homogenous and fixed-effects analysis was conducted. When I^2^ > 50%, studies were considered to have been heterogeneous and random-effects analysis was conducted. If the heterogeneity between study groups was too great, descriptive analysis was performed. Publication bias was analyzed using funnel plots. The impact of each study on pooled OR was assessed case-by-case.

## 3. Results

### 3.1. Literature search and eligible studies

**Figure 1:**
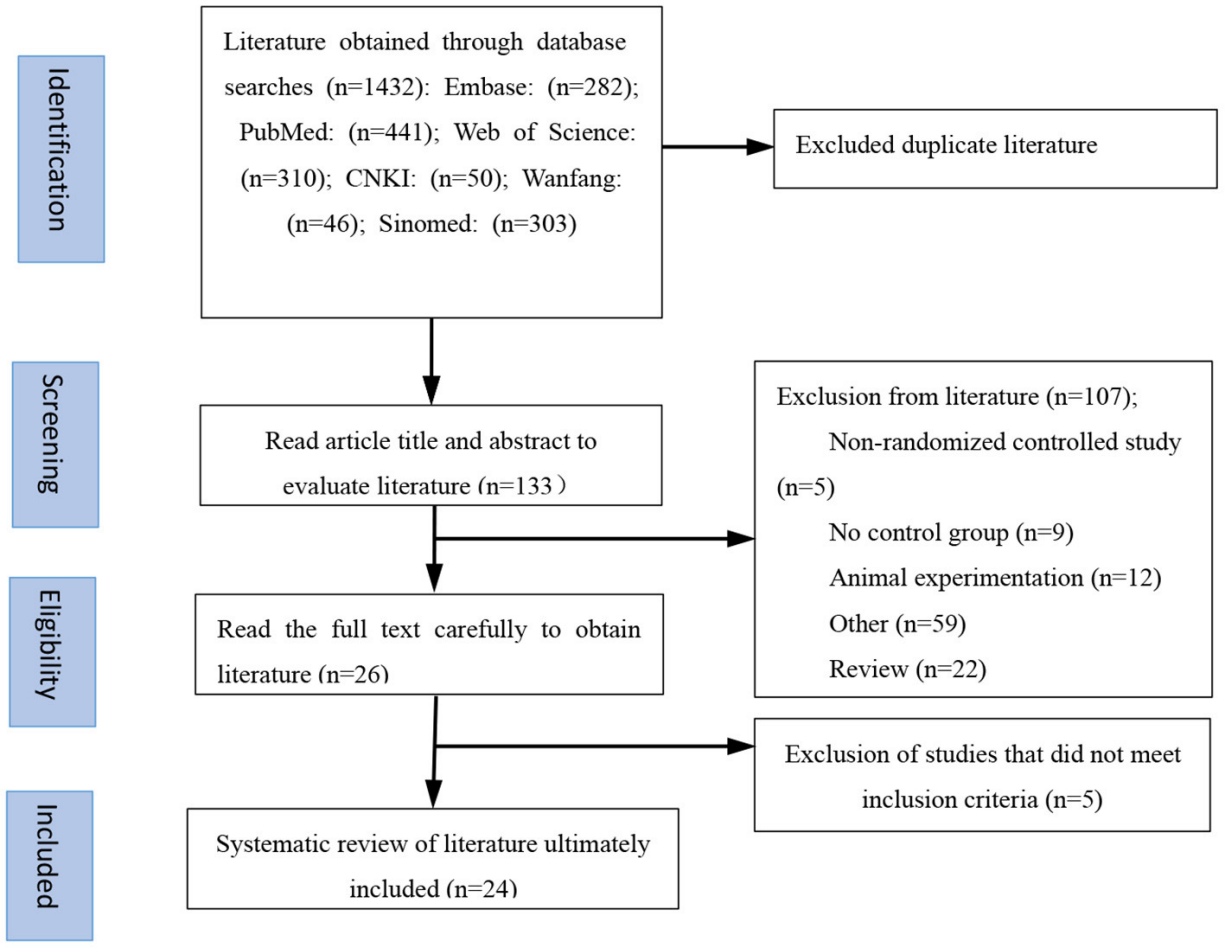
Flow chart for systematic screening of literature.

The flow chart in Figure 1 details the process of literature screening used in this study. After elimination of duplicate publications, our initial screening identified a total of 133 potential studies. After reading the study titles and abstracts, 107 studies were excluded either because they lacked a control group, were animal studies, were duplicate publications, or were conference papers. The remaining 26 studies were assessed in more detail and data from five studies were determined to not meet inclusion criteria. The reasons for excluding these five studies from our analysis included a lack of detailed genotype documentation, reports of patients having suffered comorbidities in addition to MDD, or clinical review or meta-analysis publication content. A total of 24 publications were ultimately included in this study. The characteristics of the included literature are detailed in Table 1; eight publications were written in Chinese, covering 4,116 Asian patients, and 16 were written in English, covering 1,662 Caucasian patients. All the literature included in this study was published after 2005 and analyzed minimum and maximum study sample sizes of 58 and 3,123, respectively. Of these 24 studies, 19 reported the Val66Met genotype in detail while the remainder combined the number of allele carriers.

**Table 1:** Characteristics of eligible literature.

**References**	**Country**	**Ethnicity**	**Sample size**	**Average Age**		**Genotype and Allele**				**Evaluation Criteria**	**HWE**
			**MDD:C**	**MDD**	**C**	**M**		**C**			
						**VV:VM:MM or VV:M-carry**	**V:M**	**VV:VM:MM or VV:M-carry**	**V:M**		
Juan et al. (16)	Spain	Caucasian	209/2,914	< 60	< 60	130:66:13	326:92	1,817:978:119	4,612:1,216	DSM-IV	Y
Maria et al. (17)	Poland	Caucasian	61/66	< 60	< 60	40:18:3	98:24	47:17:2	111:21	DSM-V	Y
Mariam et al. (18)	United States	Caucasian	45/45	47.1 ± 17.8	40.4 ± 17.7	21:22:2	64:26	34:10:1	78:12	DSM-IV	Y
Yujin Lee et al. (19)	Republic of Korea	Asian	209/101	62	61	62:102:45	226:192	23:62:16	108:94	DSM-IV	Y
Amy Froud et al. (20)	Australia	Caucasian	121/48	< 60	< 60	81:38:2	200:42	34:13:1	81:15	DSM-V/DSM-IV	Y
You et al. (21)	China	Asian	99/110	69.69 ± 5.38	69.45 ± 5.14	25:48:26	98:100	26:59:25	111:109	DSM-IV	Y
Dandan Lou et al. (22)	China	Asian	110/100	< 60	< 60	31:56:23	118:102	24:49:27	97:103	DSM-IV	Y
Ying and Yuqi (23)	China	Asian	106/175	65.92 ± 6.11	64.87 ± 7.42	39:43:24	121:91	48:86:41	182:168	CCMD-3	Y
Ai et al. (24)	China	Asian	125/91	42.9 ± 16.1	41.2 ± 15.5	30:66:29	126:124	13:52:26	78:104	CCMD-3/DSM-IV	-
Shen et al. (25)	China	Asian	105/111	18–45	18–45	34:54:17	122:88	33:59:19	125:97	HDRS ≥ 18	Y
Frodl et al. (26)	Germany	Caucasian	60/60	44.2 ± 11.8	41.6 ± 12.3	37:21:2	95:25	40:19:1	99:21	DSM-IV	Y
Warren et al. (27)	United States	Caucasian	245/94	69.7 ± 7.5	69.8 ± 5.8	150:85:10	385:105	71:22:1	164:24	-	Y
Yu (28)	China	Asian	32/49	15.5 ± 1.5	15.7 ± 1.2	7:17:8	31:33	16:21:12	53:45	DSM-V	Y
Xiaorui (29)	China	Asian	41/44	< 60	< 60	13:20:8	46:36	14:21:9	49:39	DSM-IV	Y
Jianyue (30)	China	Asian	396/324	18–60	18–60	107:189:100	403:389	88:163:73	339:309	DSM-IV	Y
Chunxia (31)	China	Asian	447/432	27.76 ± 7.98	28.32 ± 8.67	89:213:121	391:455	95:210:118	400:446	DSM-IV	Y
Yin et al. (32)	China	Asian	26/33	≥60	≥60	8:18	-	9:24	-	DSM-IV	Y
Sophyia et al. (33)	United States	Caucasian	176/88	68.07 ± 7.02	70.13 ± 5.60	104:72	-	67:21	-	-	-
Dora et al. (34)	United States	Caucasian	33/23	≥60	≥60	17:16	-	12:11	-	DSM-IV	Y
Warren et al. (35)	United States	Caucasian	199/113	70.0 ± 7.8	69.9 ± 5.6	120:79	-	83:30	-	DSM-IV	Y
Ozan et al. (36)	Turkey	Caucasian	66/56	< 60	< 60	36:30	-	38:18	-	DSM-IV	-
Haldar et al. (37)	India	Asian	104/106	< 60	< 60	19:59:26	97:111	24:62:20	110:102	ICD-10	Y
Kreinin et al. (38)	Israel	Caucasian	51/38	< 60	< 60	31:18:2	80:22	22:15:1	59:17	DSM-IV	-
Sun et al. (39)	China	Asian	431/402	< 60	< 60	128:215:88	471:391	107:202:93	416:388	DSM-IV	Y

MDD, major depressive disorder group; C, control group; Y, yes; HWE, Hardy-Weinberg equilibrium; DSM-IV, Diagnostic and Statistical Manual of Mental Disorders IV; DSM-V, Diagnostic and Statistical Manual of Mental Disorders V; CCMD-3, Chinese Classification and Diagnostic Criteria of Mental Disorders-3; HAMD, the Hamilton Depression Rating Scale; ICD-10, International Classification of Diseases-10.

### 3.2. Quantitative analysis

**Figure 2:**
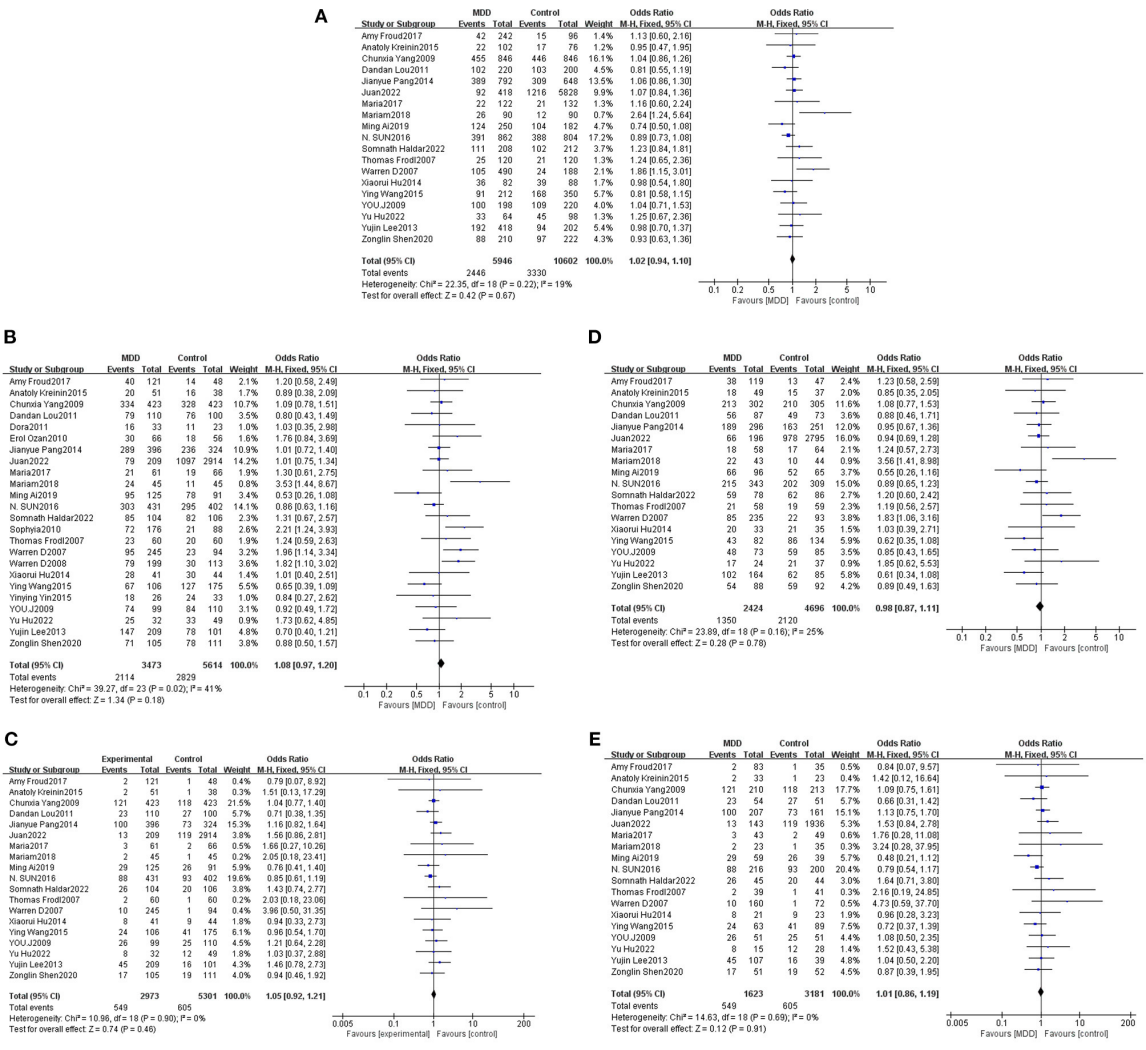
Forest plots of the association of Val66Met with MDD [**(A)** allele model; **(B)** dominant model; **(C)** recessive model; **(D)** heterozygous model; **(E)** homozygous model].

Total effect analysis of the Val66Met genetic model using heterogeneity testing revealed *P* > 0.05 and I^2^ ≤ 50% for all genetic models, suggesting that there was little heterogeneity between the studies and all fixed-effects models were adopted for analysis. Analysis of Met allele data revealed a Val OR of 1.02 (95% CI: 0.94, 1.10), confirming that there was no statistically significant difference between Met and Val alleles in MDD patients as compared to the healthy population. Meta-analysis findings based on different genetic models revealed dominant, recessive, heterozygous, and homozygous gene model ORs to be 1.08 (95% CI: 0.97, 1.20), 1.05 (95% CI: 0.92, 1.21), 0.98 (95% CI: 0.87, 1.11), and 1.01 (95% CI: 0.86, 1.19), respectively, confirming that differences between MDD patients and healthy controls were not statistically significant for each gene model. Thus, no significant association of the Val66Met polymorphism with MDD was determined (Table 2, Figure 2).

**Table 2:** Meta-analysis of the association between Val66Met and MDD.

**Val66met**	**Dominant model (MM**+**VM:VV)**		**Recessive model (MM:VV**+**VM)**		**Heterozygous model (VM:VV)**		**Homozygous model (MM:VV)**		**Allele models (M:V)**	
	***P*** **value**	**OR (95%)**	***P*** **value**	**OR (95%)**	***P*** **value**	**OR (95%)**	***P*** **value**	**OR (95%)**	***P*** **value**	**OR (95%)**
Overall	0.18	1.08 [0.97, 1.20]	0.46	1.05 [0.92, 1.21]	0.78	0.98 [0.87, 1.11]	0.91	1.01 [0.86, 1.19]	0.67	1.02 [0.94, 1.10]
Caucasian	0.0001	1.40 [1.18, 1.66]	0.03	1.70 [1.05, 2.78]	0.10	1.20 [0.97, 1.48]	0.02	1.77 [1.08, 2.88]	0.01	1.25 [1.05, 1.48]
Asian	0.16	0.91 [0.79, 1.04]	0.85	1.01 [0.88, 1.17]	0.14	0.89 [0.77, 1.04]	0.53	0.95 [0.79, 1.13]	0.46	0.97 [0.89, 1.06]
Age < 60	0.58	1.04 [0.90, 1.20]	0.80	1.02 [0.88, 1.19]	0.99	1.00 [0.87, 1.15]	0.92	1.01 [0.84, 1.21]	0.77	1.01 [0.93, 1.11]
Age ≥ 60	0.41	1.17 [0.80, 1.72]	0.21	1.24 [0.88, 1.75]	0.63	0.88 [0.51, 1.50]	0.96	1.01 [0.68, 1.51]	0.63	1.08 [0.79, 1.46]

**Figure 3:**
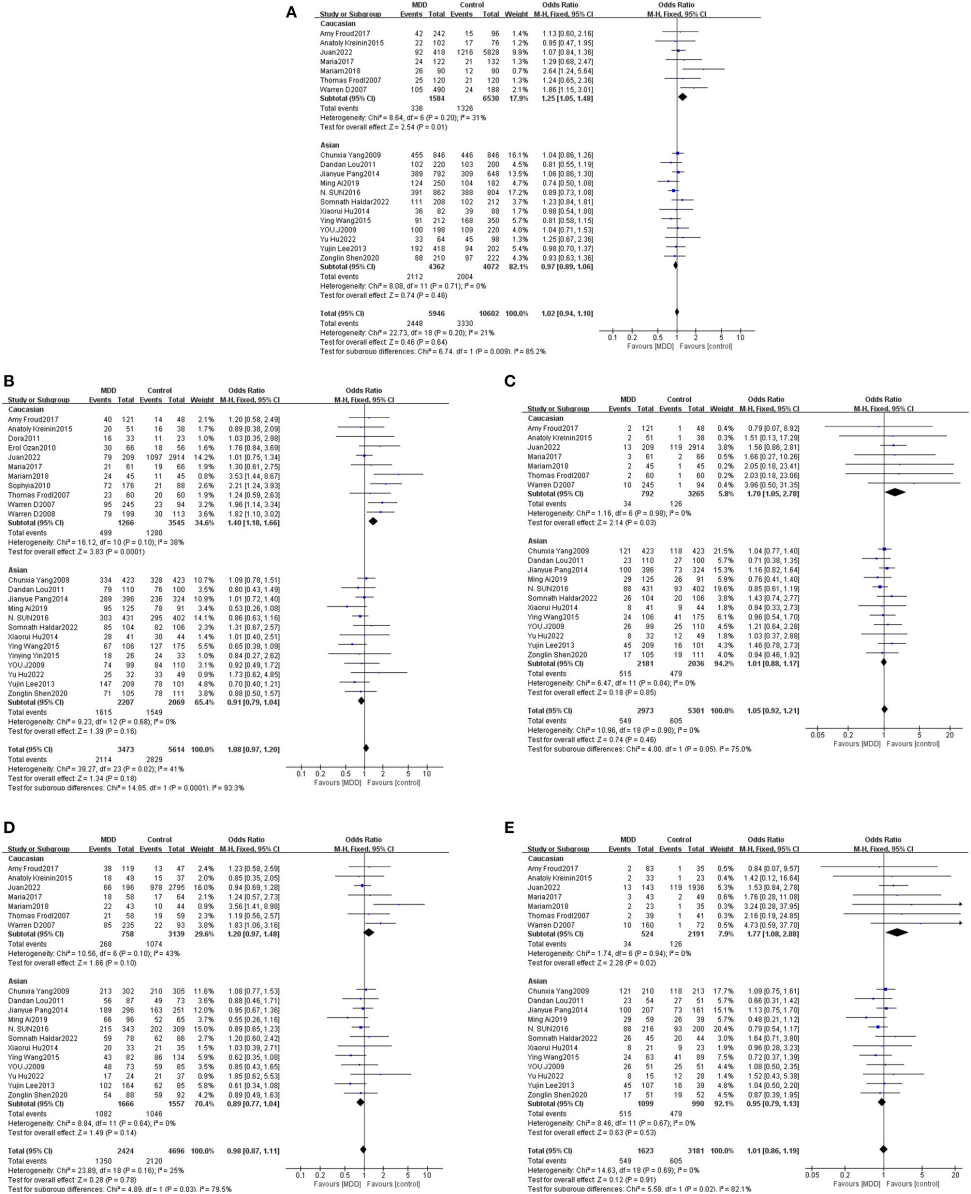
Forest plots of the association of Val66Met with MDD according to ethnicity [**(A)** allele model; **(B)** dominant model; **(C)** recessive model; **(D)** heterozygous model; **(E)** homozygous model].

Ethnicity-based subgroup analysis of the BDNF Val66Met gene model using heterogeneity testing revealed *P* > 0.05 and I^2^ ≤ 50% for all gene models; fixed-effects models were adopted for analysis. The results revealed an OR of 1.25 (95% CI: 1.05, 1.48, *P* = 0.01) for the Caucasian allele Met:Val, suggesting that Val allele frequency was reduced while Met allele frequency was increased in white MDD patients as compared to controls. Thus, the Met allele was found to be associated with a genetic susceptibility to MDD among white populations. Meta-analysis based on different genetic models revealed dominant, recessive, heterozygous, and homozygous gene model ORs for white populations to be 1.40 (1.18, 1.66, *P* = 0.0001), 1.70 (1.05, 2.78, *P* = 0.03), 1.20 (0.97, 1.48, *P* = 0.10), and 1.77 (1.08, 2.88, *P* = 0.02), respectively. Statistically significant differences between dominant, recessive, and homozygous gene models were found. These three gene models were found to be associated with a genetic susceptibility to MDD in Caucasian populations. The results of the Asian populations subgroup meta-analysis were not statistically significant and consequently the Val66Met polymorphism was not determined to associate with a genetic susceptibility to MDD in Asian populations (Table 2, Figure 3).

**Figure 4:**
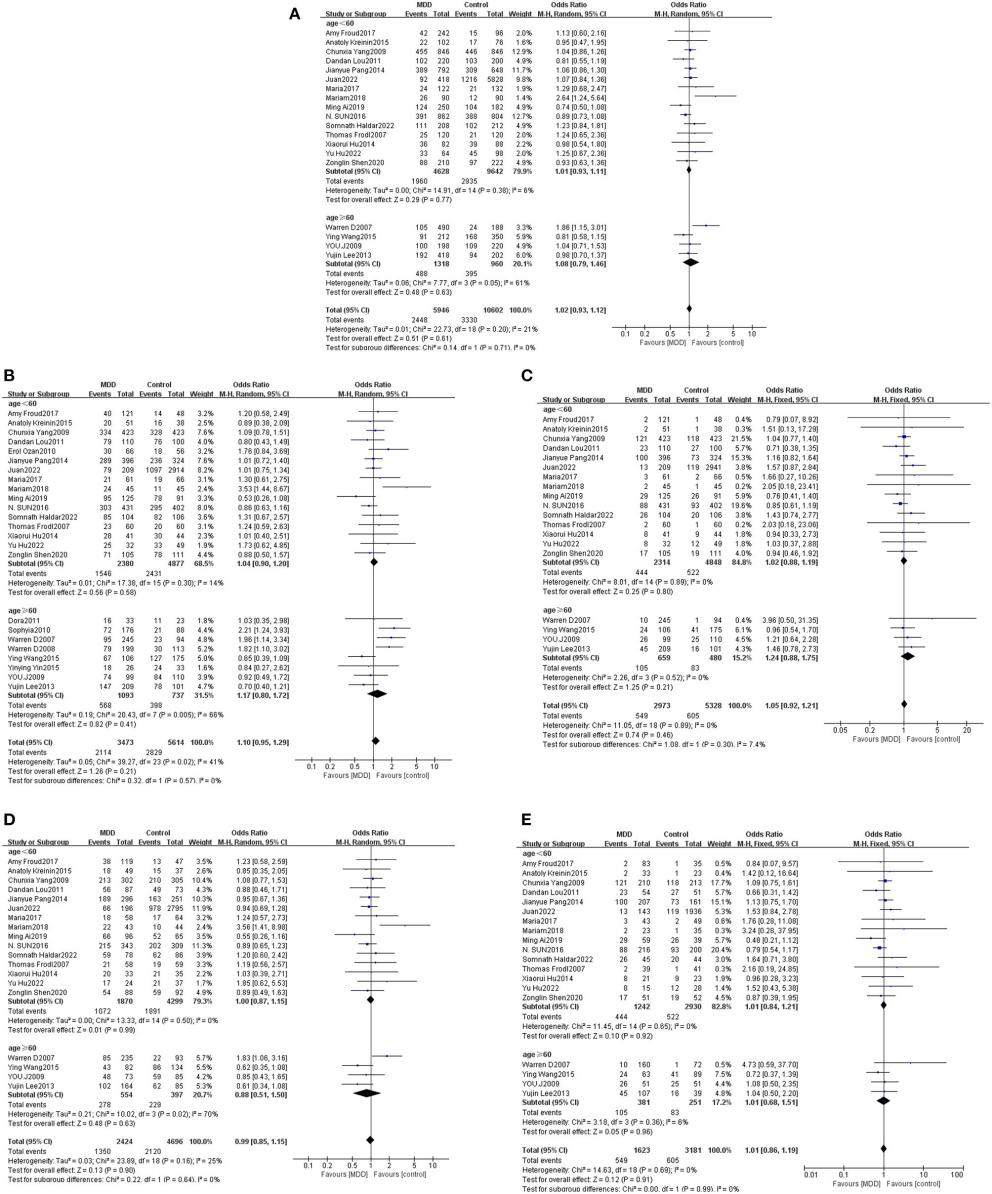
Forest plots of the association of Val66Met with MDD according to age [**(A)** allele model; **(B)** dominant model; **(C)** recessive model; **(D)** heterozygous model; **(E)** homozygous model].

According to the changes in the physiological and psychological structure of modern people, the World Health Organization (WHO) has defined age categories for people, among which elderly people are considered to be those over 60 years old. Age-based subgroup analysis was performed considering 60 years of age as a cut-off for dividing the subjects into two groups. Heterogeneity testing revealed that *P* < 0.05 and I^2^ > 50% for alleles, dominant genes, and heterozygous genes in subjects older than 60 years; a random-effects model was thus employed for this population while a fixed-effects model was used for analysis of the remaining gene models with I^2^ ≤ 50%. Meta-analysis results revealed no statistically significant differences between Met and Val alleles. As such, no statistically significant differences relevant to Met and Val alleles between MDD patients of different ages as compared with healthy persons were found. Thus, age was determined not to be associated with the BDNF Val66Met gene polymorphism or genetic susceptibility to MDD (Table 2, Figure 4).

### 3.3. Assessment of sensitivity and publication bias

**Figure 5:**
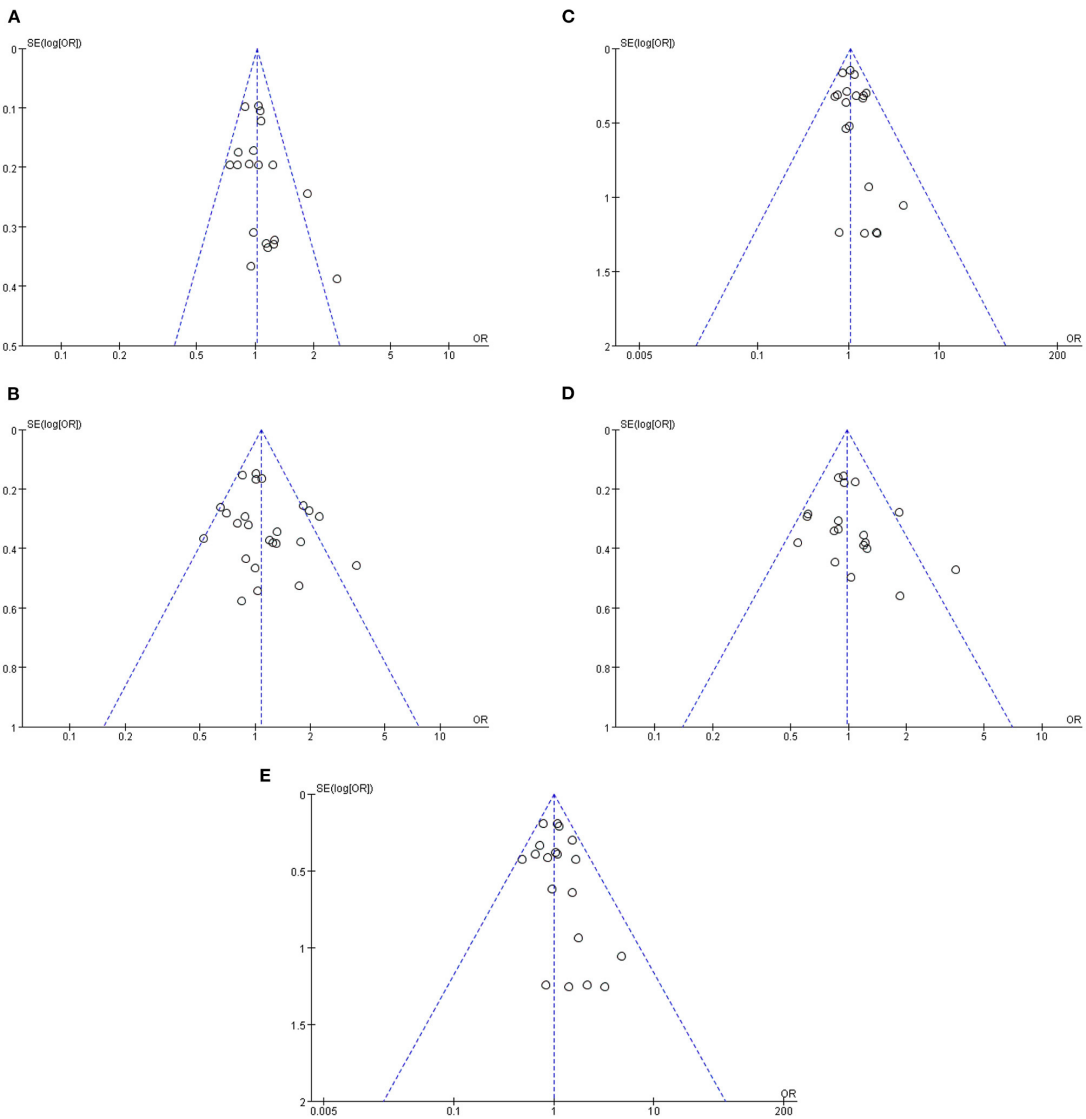
Funnel plots of the association of Val66Met with MDD [**(A)** allele model; **(B)** dominant model; **(C)** recessive model; **(D)** heterozygous model; **(E)** homozygous model].

Sensitivity analysis revealed that risk estimates were not significantly affected upon exclusion of any individual study, confirming that the results of this meta-analysis were reliable. According to genotype indicator, a funnel plot analysis of potential publication bias revealed a roughly symmetrical distribution of values and no significant pattern asymmetry, suggesting that the publication bias in the literature we analyzed was negligible (Figure 5).

## 4. Discussion

The pathophysiological mechanisms via which the BDNF Val66Met gene polymorphism acts to induce MDD remain unclear. A dimeric, neurotrophic factor primarily concentrated in the hippocampus and frontal cortex, BDNF is closely related to neuronal plasticity, differentiation, survival, and repair (20). Importantly, BDNF exerts beneficial effects in the setting of depressive and anxiety disorders (40). The role of neurotrophic factor BDNF in psychopathology has attracted more and more attention, and it may be one of the biomarkers of MDD. There are three haplogroups of the BDNF Val66Met polymorphism: Val/Val, Val/Met, and Met/Met. Animal studies have suggested that this polymorphism is associated with a genetic susceptibility to anxiety and depression. Furthermore, this polymorphism was reported to be associated with the prevalence of depression in a population (41, 42). Musazzi (43) exposed adult male BDNF Val/Val and BDNF Val/Met knock-in mice to 30 min of acute restraint stress. Interestingly, BDNF Val/Met mice exhibited higher levels of presynaptic glutamate release and hippocampal cAMP response element binding protein (CREB) phosphorylation as compared to BDNF Val/Val mice, suggesting that Met carriers are more vulnerable to stressful events. The Val66Met polymorphism is a major genetic influence on brain development and neuronal plasticity (44). Cattaneo et al. found significantly lower BDNF protein levels in Met carriers as compared to the Val/Val genotype, suggesting that this polymorphism plays an important role in individual differences in susceptibility to mental illness (45). Bueller reported a significantly increased susceptibility to various neuropsychiatric disorders among M allele carriers. Heterozygous populations carrying the M allele have reduced hippocampal volume and impaired learning memory. Compared to the VV genotype, M carriers were reported to have impaired situational memory, a common symptom in MDD (46). Other studies, however, concluded the opposite. Sen et al. studied a healthy population using the personality factor scale and found that trait anxiety and neuroticism scores were higher in VV genotypes as compared to M allele carriers, suggesting VV populations to be more prone to depression (47). A meta-analysis of the Val66Met gene polymorphisms revealed no statistically significant differences between healthy and depressed persons (48). Although findings reported by Gyekis revealed that the BDNF Val66Met gene polymorphism was not associated with susceptibility to depressive disorders, no further subgroup analysis was performed in that study and its findings thus lacked comprehensiveness. Thus, it is necessary to further investigate whether BDNF Val66Met polymorphism is associated with MDD susceptibility.

The literature used in our study on Caucasian populations included 11 articles, five from the United States and remaining six from Poland, Spain, Turkey, Germany, Israel, and Australia. The literature on Asian populations included 13 articles, 11 of which were from China and one each from the Republic of Korea and India. In this work, we retrieved 24 studies including 3,497 cases and 5,623 controls, thus studying a relatively large sample size that certainly reduced the probability of false negatives. Our findings revealed that the total effect of the BDNF Val66Met gene polymorphism was not significantly associated with MDD. However, a subgroup analysis revealed that the Met allele was associated with a genetic susceptibility to MDD in Caucasian populations, with statistically significant differences between dominant, recessive, and homozygous genotypes. Age was not found to associate with any susceptibility to MDD. No publication bias was noted in our study.

The present study suggests BDNF Val66Met gene polymorphism plays a role in major depressive disorder in Caucasian populations but no such pattern was found in Asian populations. We considered that the possible reasons for the conflicting results include changes in the frequency of the same gene mutation between populations, the impact of the same gene mutation on different races and ages, and the impact of different environments. The frequency of Met alleles in white populations is 25–32% (15), while the frequency of Met alleles in Asian populations is more frequent, about 40–50% (49, 50). There are differences in the lifetime prevalence of major depression between Western and Asian populations, with Asian populations having a lower lifetime prevalence of major depression. A cross-country epidemiological survey showed that the lifetime prevalence of MDD was 1.5% in Taiwan and 2.9% in the Republic of Korea (51), compared with an average of 15–17% for Western populations (52, 53). The high frequency of Met alleles in Asian populations is present in a lower prevalence of major depression, which may indicate that Met alleles protect against major depression. As the M allele occurs more frequently in Asian populations, low frequencies of the V allele and VV genotype likely weakened the association between the polymorphism and susceptibility to depression reported in prior studies (54). Zarza-Rebollo et al. (16) genotyped the BDNF Val66Met gene polymorphism in 3,124 people in the community, 209 of whom suffered from depression, and the incidence of MDD decreased under the interaction between Met allele carriers and the environment. Maria Skibinska (17) and Froud et al. (20) showed that the BDNF Val66Met gene polymorphism was not associated with BDNF serum levels, and this polymorphism did not seem to predict BDNF levels or the incidence of depression. However, Youssef et al. (18) and Taylor et al. (27) noted that Met carriers had a higher risk of depression, and Met allele carriers were almost twice as likely to develop depression in old age compared with pure-allele carriers of Val allele. Frodl et al. (26) suggested that Met allele carriers may be at risk of developing smaller hippocampal volumes and may have implications for vulnerability to MDD. Kanellopoulos et al. (34) believed that Met carriers develop depression at an earlier age than Val/Val homozygotes. Some studies have shown that the relationship between Val66Met polymorphism and MDD may be influenced by environmental factors; for example, negative childhood life events may moderate the relationship between Val66Met gene polymorphism and MDD, with a stronger association observed in individuals who have experienced negative life events (55). In addition, some environmental factors may influence the level and function of BDNF, which is related to the expression and function of Val66Met gene. For example, exercise, antidepressant medication, and dietary fat intake have been shown to influence BDNF level and function (56). Thus, future studies with larger Caucasian sample sizes are required to confirm our findings.

This meta-analysis has some limitations. Since our study only included literature published in Chinese and English, the possibility of publication bias cannot be ruled out. As the number of available case-control studies was relatively small, future meta-analyses evaluating larger samples are required to draw more accurate conclusions. Due to limited data, we were unable to stratify analysis according to factors such as years of education, sex, and exposure to various environmental factors. More precise assessments of the literature should be conducted in future meta-analyses.

## Data availability statement

The original contributions presented in the study are included in the article/supplementary material, further inquiries can be directed to the corresponding author.

## Author contributions

NL, ZZ, and ZS were responsible for the literature collection and collation. YW and OL processed the data and wrote the paper. JX revised the article. All authors contributed to the article and approved the submitted version.
